# Study on soil erosion and its driving factors from the perspective of landscape in Xiushui watershed, China

**DOI:** 10.1038/s41598-023-35451-7

**Published:** 2023-05-20

**Authors:** Linsheng Wen, Yun Peng, Yunrui Zhou, Guo Cai, Yuying Lin, Baoyin Li

**Affiliations:** 1 State Key Laboratory for Subtropical Mountain Ecology, Ministry of Science and Technology and Fujian Province, Fujian Normal University, Fuzhou, 350117 China; 2 School of Geographical Sciences, School of Carbon Neutrality Future Technology, Fujian Normal University, Fuzhou, 350117 China; 3 Postdoctoral Research Station of Ecology, Fujian Normal University, Fuzhou, 350117 China; 4 School of Culture, Tourism and Public Administration, Fujian Normal University, Fuzhou, 350117 China; 5 The Higher Educational Key Laboratory for Smart Tourism of Fujian Province, Fuzhou, 350007 China; 6 Yuanzhou District Forestry Bureau, Yichun, 336000 Jiangxi China

**Keywords:** Ecosystem services, Macroecology, Natural hazards

## Abstract

Soil erosion (SE) is one of the most serious disasters in the world, which directly damage the productivity of the land and affect human well-being. How to effectively mitigate soil erosion is a challenge faced by all countries in the world. In this study, soil erosion was quantitatively assessed base on the RULSE model in an ecologically fragile area [Xiushui watershed (XSW)], and the effects of three major categories of factors (land use/cover change, landscape fragmentation and climate) on soil erosion were investigated using correlation analysis and structural equation model. The results indicated that there was no continuous increase or decrease trend on the SE of XSW with impact of rainfall, the mean values of SE were 2205.27 t/ha, 3414.25 t/ha and 3319.44 t/ha from 2000 to 2020 and the hot areas of SE were mainly distributed around the Xiushui river channel, respectively. The expansion of urbanization (the area of impervious increased from 113.12 to 252.57 km^2^) aggravated landscape fragmentation, and the landscape fragmented area had some overlap with the hot zone of SE. Additionally, the LUCC factor dominated by NDVI, landscape fragmentation factor and climate factor dominated by rainfall had a directly driving effect on SE, where the path coefficient of landscape fragmentation was 0.61 (*P* < 0.01), respectively. We also found that except increasing forest area, improving forest quality (NDVI, canopy closure, structure) deserved emphasized in SE management, and the effect of landscape fragmentation on SE also should not be ignored. Moreover, soil erosion assessment at large scales over long time periods tends to underestimate the driving force of rainfall on SE, and it is a great challenge to evaluate the effect of extreme rainfall on soil erosion at short time scales in a downscale manner. This research provides insights for ecological sustainable management and soil erosion protection policies.

## Introduction

Soil erosion is a common natural disaster and occurs all over the world^1–3^. It is a process of soil degradation manifested cause by the loss of soil nutrients and the movement of fine particles, which mainly arise from the washing of rainfall and the particles exchanging in soil–water interface^4,5^. According to statistics at the end of the last century, global agricultural land (more than 30%) was suffered different levels of soil erosion, in addition, the rate of soil loss continuing exceeds 10 million ha/a^3,6^. Moreover, SE also have always been a major threat to food security, ecosystem regulation and the well-being of mankind, because the speed required for soil development and formation is many orders of magnitude higher than soil erosion^7^. Meanwhile global governments and environmental protection agency are eager badly to address the issue of SE, and they generally start to tackle it by some ecologically fragile areas, degraded red soil areas and hilly areas with a lot of rainfall^7–10^. However, the assessment and mapping of SE are crucial for identifying the poor areas of SE and solving this predicament, meanwhile, it is also the foundation for the implementation of ecological restoration strategies.

Currently, the research methods for soil erosion assessment mainly consist of classical field measurements and the utilization of empirical models combined with GIS to assess the amount of soil erosion in a region^7,8,11^. On-site measurements (classical field measurements), which were rather a narrower range measurements on selected points, were often constrained to agricultural irrigation experiments or sediment transport in small catchment^12,13^. Model assessments to quantify soil erosion were widely used at large scales and were less labor-intensive and costly^3,4,7^. Model assessments has become a fairly popular and scientific method of soil erosion assessment for current research^9,14^, although classical field measurements are still fundamental and indispensable^13^.

Previously, numerous quantification models have been open up to evaluate SE, including the USLE (the universal soil loss equation)^15^, the RUSLE (the revised universal soil loss equation)^16^, the ZQ ( the Zengg equation)^17^, Sediment delivery ratio module of In-VEST model^18^, the USPED (the unit stream power-based erosion deposition)^19^, and the European soil erosion model (EUROSEM)^11,20^. Among these models, the most widely used were the USLE and RUSLE models^15,16^, which had widely applied in areas of different scales and environmental conditions due to their simplicity, lower entry threshold and high precision^2,13,21^.

A lot of research work has been done on SE assessment^22^, which was divided into three categories: (a) scholars had focused on different research scales to assess the risk of soil erosion, including global^23^, national^24^, urban^25^, and watershed scales^9,14,21^; (b) studies that combined soil erosion with other ecosystem services (ESs), for instance, Gong et al.^10^ explored the trade-off/synergies between soil conservation and other ESs in the mountainous basin and Geng et al.^14^ explored the current and future trends of ESs in the Yellow River basin by combining SE and water production, etc.; (c) using model simulations to predict future soil erosion risk, such as Liu et al.^9^ obtained future soil erosion characteristics based on the Dinamica environment for geoprocessing objects (EGO) for land use simulation and used them as a basis for ecological policy. However, it is not enough to spatially map the sensitive areas of SE and implement soil erosion remediation. Correctly understanding the key drivers of soil erosion in an area is more relevant for the regulation of soil erosion risk^7,26^.

Soil erosion is influenced by many factors, including land use/cover change (LUCC)^9^, geographical conditions (topography, slope length and steepness)^27^, soil physical and chemical properties (soil particle composition, structured and infiltration properties)^28^ and rainfall^29^, etc. Among these factors, the contribution of slope to soil erosion could over 60% (Redundancy analysis)^30^, but slope, soil texture and other subsets are accompanied by properties that cannot be easily changed. On the contrary, factors such as rainfall and LUCC are variable factors that are susceptible and accompanied by uncertainty^31^, which is a more crucial part that should be paid more attention to when formulating ecological restoration policies^26,32^. In previous researches, the intensity of rainfall could significantly increase soil erosion, and the area of forest was also a key factor in determining the level of soil erosion risk^2,29^. However, little attention has been paid to that landscape fragmentation is also an important factor that has an impact on ecological function^26,33^. Therefore, in this study, we classified forest cover, Normalized Difference Vegetation Index (NDVI) as LUCC factors, rainfall, evaporation and temperature were selected as climate factors^34^, and selected indicators that representing landscape fragmentation as landscape factors^26^, then used statistical methods to investigate the driving force of the factors on soil erosion. It could fill part of the research gap on soil erosion driving mechanism.

Xiushui watershed (XSW) located in the south bank reach of the Yangtze River, with abundant water and heat resources that have created extensive forests and arable land, and is an important forestry and grain resource storage area in China^35^. However, the expansion of urbanization and the increased frequency of extreme weather in recent decades had led to dramatic changes in land types and severe ecological degradation^36^. In addition, intensive agricultural intensification and social pressure greatly increase the risk of SE^8^. The implementation of ecologically sustainable management of the XSW is urgent to prevent the continued degradation of the ecosystem. A mapping assessment of SE in the XSW could provide an effective aid to the formulation of ecological management policies. Meanwhile, identifying which factor (land cover, landscape fragmentation, climate) drive soil erosion in this ecologically fragile area could help take more effective control measures and also provide a more scientific reference. In this study, we had three main objectives: (1) revealing the variations of LUCC and landscape fragmentation in the XSW from 2000 to 2020; (2) assessing the SE characteristic in XSW from 2000 to 2020; (3) revealing the key drivers of SE in XSW based on statistical analysis.

## Data sources and methodology

### Study area

**Figure 1:**
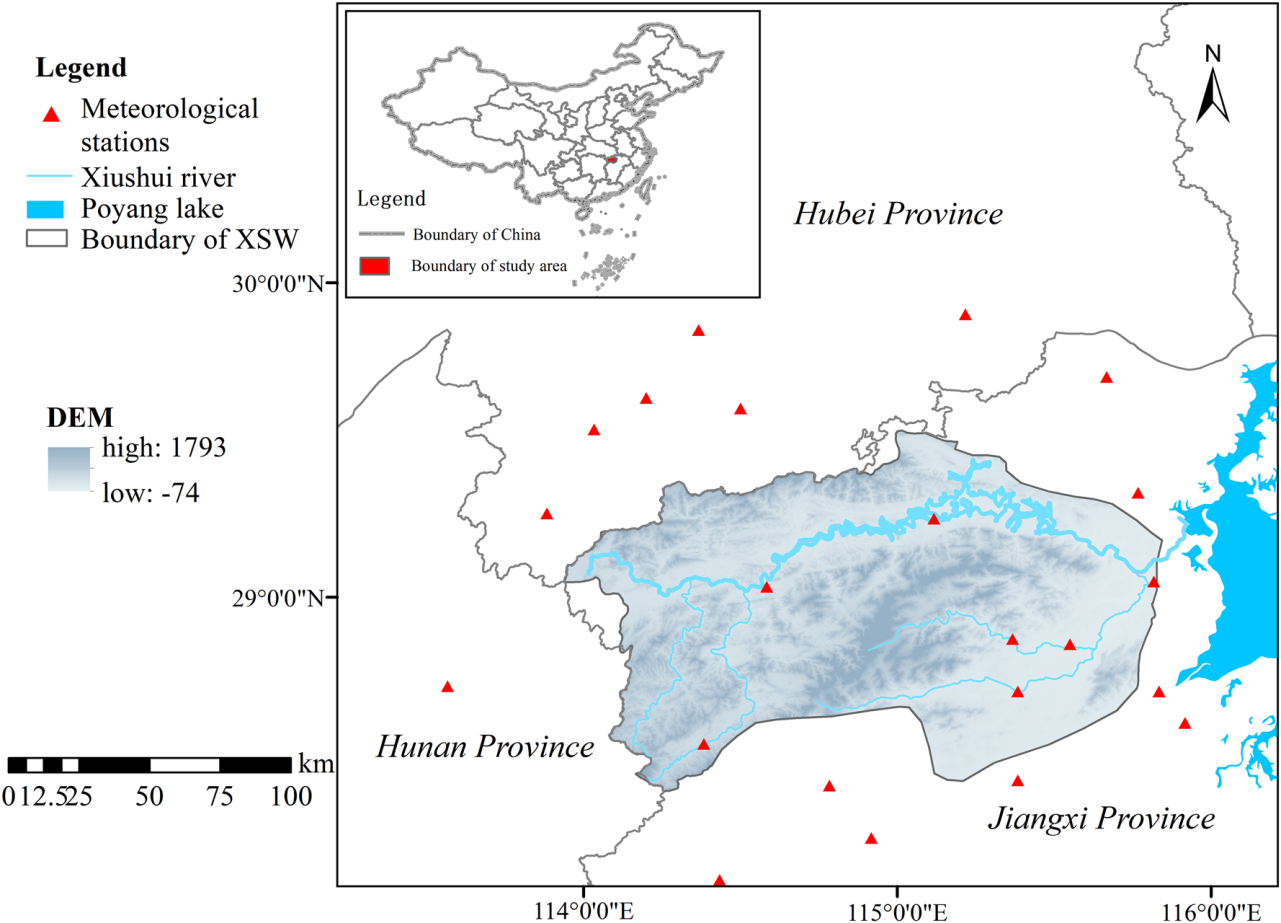
The geographic position, elevation of the study region generated in the ArcGIS 10.5 software (https://www.esri.com).

**Figure 2:**
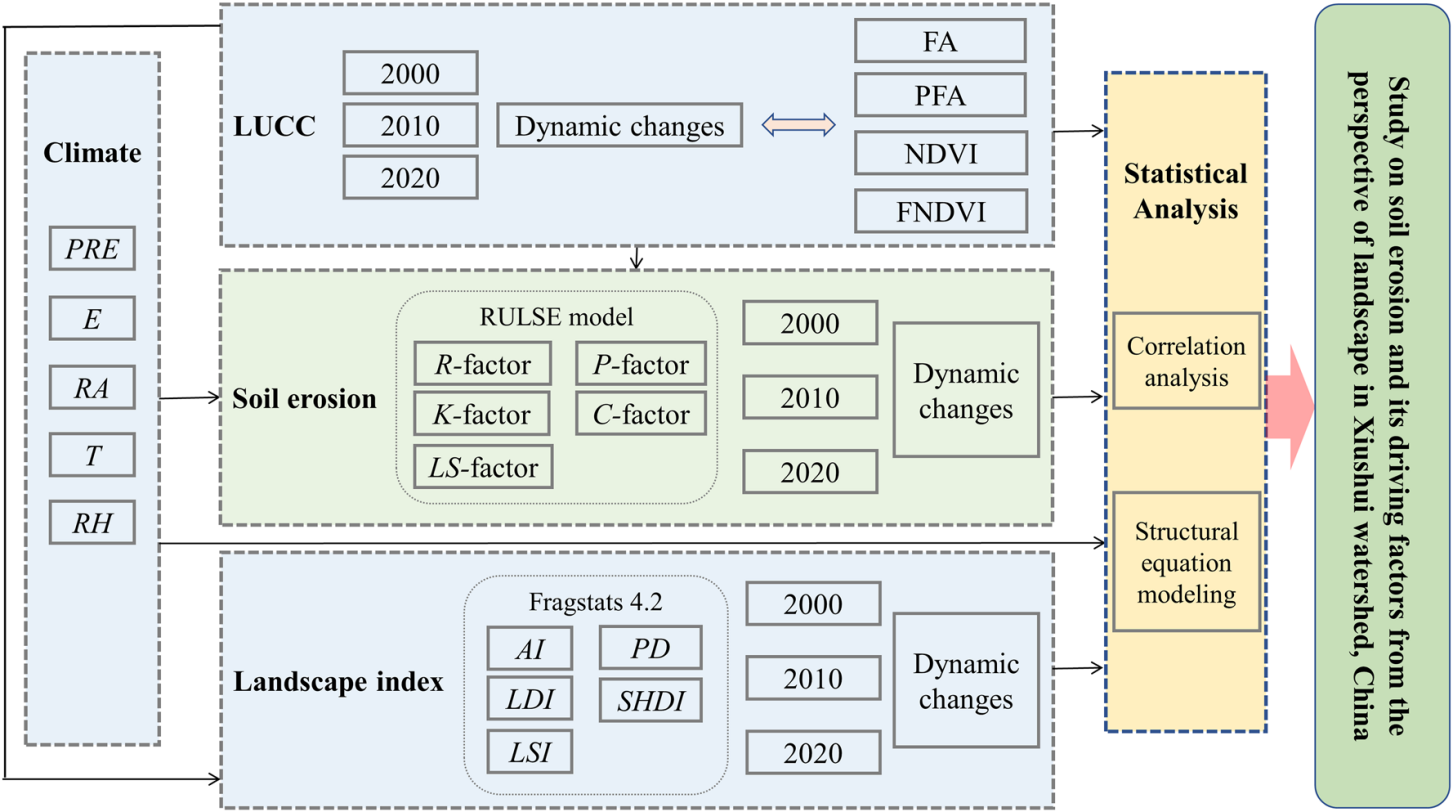
Methodological approach and sequence. The area of forest (FA), the percentage of forest area (PFA), normalized difference vegetation index (NDVI), forest contribution to NDVI (FNDVI); precipitation (PRE), evaporation (E), radiation (RA), temperature(T), relative humidity(RH); the rainfall–runoff factor (*R-*factor), the soil erodibility factor(*K-*factor), the slope length and steepness factor (*LS-*factor), the vegetation coverage factor (*C-*factor), the water and soil conservation measure factor (*P-*factor); aggregation index (AI), landscape division index (LDI), landscape shape index(LSI), patch density (PD), Shannon's diversity index (SHDI).

Xiushui watershed (XSW) in northern Jiangxi Province, China (28°22′44"–29°31′26" N, 113°56′35"–115°50′45" E), is an ecologically fragile watershed located in the south bank reach of the Yangtze River, with an area 14.79 thousand km^2^. The northwest and southwest of the XSW are the Makufu mountains and the Jiuling Mountains, which are its main forest distribution areas, and the east is close to the largest freshwater lake in China (Poyang Lake) (Fig. 1)^37^. XSW belongs to a subtropical monsoon climate region, which the average annual precipitation is 1681 mm and annual temperature (multiyear) of 17.5 ℃. The rainfall pattern in the region could be clearly distinguished between the rainy and dry seasons, and over two-thirds of rainfall occurs in April to September^36^. Accelerated urbanization, intensive agricultural operations and sufficient rainfall had led to increased erosion risk and lots of ecological degradation in the XSW. Therefore, we conducted a dynamic evaluation of soil erosion and landscape indices in XSW, and combined statistical analysis methods to explore the driving factors of soil erosion (Fig. 2).

### Data source and processing method

Land use/cover change (LUCC) maps, digital elevation model (DEM), soil properties, Normalized Difference Vegetation Index (NDVI) and meteorological dataset were applied to assess soil erosion based on RULSE^9^. LUCC in 2000, 2010 and 2020 that according to “Chinese Classification Criteria of Current Land Use” (GB/T21010-2007), the annual NDVI spatial distribution dataset in 2000, 2010 and 2020 with 30 m resolution was generated by using the maximum value synthesis method base on monthly data obtained from National Ecological Science Data Center (http://www.nesdc.org.cn) and DEM (30 m resolution) were obtained from Chinese Academy of Sciences (http://www.gscloud.cn). Soil properties data with 1 km spatial resolution (Harmonized World Soil Database version 1.1) and meteorological data that includes 22 monitoring stations in and around XWS with daily resolution were obtained from the National Cryosphere Desert Data Center (https://www.ncdc.ac.cn) and China Meteorological Administration (http://data.cma.cn), respectively.

### Quantifying soil erosion

The quantitative assessment and mapping of SE was based on the RULSE model (Eqs. 1,2 and 3)^16^, which consisted *R*-factor (rainfall–runoff, unit: MJ mm/(ha h year)), *K*-factor (the soil erodibility factor, unit: t ha h/(ha MJ mm)), *L*-factor (slope length factor), *S*-factor (steepness factor), *C*-factor (the vegetation coverage factor), *P*-factor (the water and soil conservation measure factor), respectively^13^.

1 $$S{E}_{a}= R\times K\times L\times S\times C\times P$$

2 $$S{E}_{p}= R\times K\times L\times S$$

3 $$SC= {SE}_{p}-{SE}_{a}$$

where *SE*_*p*_, *SE*_*a*_ and *SC* are the assessed potential SE per year (t/(ha year)), the estimated actual SE per year (t/(ha year)) and the assessed soil conservation per year (t/(ha year)), respectively.

1. *R*-factor: it reflects the washing effect of rainfall and runoff on SE. In the research, the calculation of *R*-factor using monthly rainfall data of observation period base on Eq. (4)^15^. The data from meteorological stations were used for interpolation based on ArcGIS tool before the calculation of *R*-factor.
  4 $$R=\sum_{1}^{12}1.735\times {10}^{(1.5{log}_{10}({P}_{i}/P)-0.0818)}$$
  where *P*_*i*_ , *P* are monthly rainfall (mm) and annual rainfall (mm), respectively.
2. *K*-factor: it indicates the sensitivity of soil particles to hydraulic scour and particle stripping^38^. And the EPIC model developed by Williams^39^ was applied to evaluate the *K*-factor as follows:
  5 $$K=0.1317\left[0.2+0.3{e}^{0.0256N\left(1-L/100\right)}\right]\times {\left(\frac{L}{A+L}\right)}^{0.3}\times \left(1.0-\frac{0.25\times TOC}{TOC+{e}^{3.72-2.95TOC}}\right)\times [1.0-\frac{0.7\left(1-L/100\right)}{(1-L/100)+{e}^{22.9\left(1-L/100\right)-5.51}}]$$
  where *N, L, A* and *TOC* represent the percentage contents of sand (0.05–2 mm), silt (0.002–0.05 mm), clay (< 0.002 mm) and soil organic carbon, respectively.
3. *L* and *S* factor: it is the steep effect of slope length and the steepness to SE. In this study, *L* and *S* were computed by using the method built up by Wischmeier and Smith^15^ and step coupling methods developed by Liu et al.^40^ and McCool et al.^41^.
  6 $$L={(\alpha /22.1)}^{\beta },\beta =\left\{\begin{array}{ll}\begin{array}{l}0.2\\ 0.3\end{array}& \begin{array}{l}\theta \le 1^\circ \\ 1<\theta \le 3^\circ \end{array}\\ 0.4& 3^\circ <\theta \le 5^\circ \\ 0.5& 5^\circ <\theta \end{array}\right.; S=\left\{\begin{array}{l}\begin{array}{ll}10.8 sin\theta +0.036& \theta \le 5^\circ \end{array}\\ \begin{array}{ll}16.8 sin\theta -0.5& 5<\theta \le 10^\circ \end{array}\\ \begin{array}{ll}21.9 sin\theta -0.96& 10^\circ \le \theta \end{array}\end{array}\right.$$
  where *α* is the non-cumulative slope length (m), *β* is the slope-length exponent, and *θ* is the slope.
4. *C*-factor: it represent the overlap effect of vegetation coverage on SE. *C*-factor was calculated with the equation developed by Gutman & Ignatov base on NDVI dataset^42^:
  7 $$C=1-\frac{NDVI-{NDVI}_{min}}{{NDVI}_{max}-{NDVI}_{min}}$$
5. *P*-factor: it is defined the ratio between the influence of contouring and tillage practices^43^, which is usually estimated base om land use/cover type^13^. We determined the *P*-factor by previous research results, combined with the similarity of the study area in this study^9,44^. (Table 1).

**Table 1:** *P* values of different land use types on the XSW.

Land use	Agricultural	Forest	Shrub	Grassland	Water	Barren	Impervious
*P* value	0.60	0.20	0.25	0.35	0.00	0.3	0.00

### Statistical analysis

The relationship among each factor and soil erosion and soil conservation were explored by the pioneer method (correlation analysis). First, 2000 variable pairs were created via sampling and zonal tools in ArcGIS from the *SE*_*a*_ grid layer and every factor (including LUCC, landscape and climate factors) to get the values of *SE*_*a*_ and its corresponding value of every factors^45,46^. Before the statistical analysis, the normal distribution of the data was checked, for which did not satisfy, we used transformation algorithms (e.g., logarithmic transformation) to improve normality^33,47^. Then, statistical analysis in R (version 4.2.1) was used to show between ES and factors. Structural equation modeling (SEM) was ultimately used to further reveal the path relationships between soil erosion and each factor.

#### LUCC, landscape fragmentation and climate factors

The factors influencing soil erosion include LUCC, forest cover and rainfall^9,22,27^. In this study, based on the results of previous scholars^22,26,33,34^, we divided the main factors affecting soil erosion into three major categories: LUCC, landscape fragmentation and climate. Landscape fragmentation and LUCC are interrelated, but the main reason for classifying "LUCC" and "landscape" factors in this study was that the landscape factor was mainly represent the landscape indexes of land fragmentation, while the LUCC factor was reflect characteristics such as the area of forest and NDVI within the grid.

1. LUCC factors: in this study, a total of four indicators the area of forest (FA, unit: ha), percentage of forest area (PFA unit: %), NDVI and forest contribution to NDVI (FNDVI) were selected to characterize the effect of land overlap on SE^13,40^. The equations of FA and FNDVI evaluate as follows:
  8 $${PFA}_{i}={FA}_{i}/{A}_{i}$$
  9 $${FNDVI}_{i}={PFA}_{i}\times {NDVI}_{i}$$
  where *i* represents the number of grids.

(B) Landscape fragmentation factors: landscape indices have a crucial impact on ESs but it usually was neglected^26^. Under the context of rapidly changing of land surface utilization, the impact of landscape fragmentation on ESs and SE cannot be ignored. Aggregation index (AI), landscape division index (LDI), landscape shape index (LSI), patch density (PD) and Shannon's diversity index (SHDI) were selected which they reflect the level of landscape fragmentation^26,33^. All these landscape index calculations were performed in Fragstats 4.2 that is a spatial analysis platform on quantifying the internal structure association of landscapes. At the landscape mosaic level, the “moving window” was conducted to calculate the landscape index based on the pixel scale and further details about the calculations could be found in the articles by Bai et al.^33^.
(C) Climate factors: rainfall was often considered to have the greatest impact on soil erosion among all climate factors^29^. However, evaporation, temperature and other factors influence the stripping of soil particles^5,48^. Therefore, as with precipitation (PRE), evaporation (E), radiation (RA), temperature (T) and relative humidity (RH) were also selected as climate factors. Details and descriptions of the above indicators can be found in Table 2.

**Table 2:** LUCC, climate and landscape fragmentation related indicators and interpretation.

Type	Indicators	Abbreviation	Description	Unit
LUCC	The area of forest	FA	The total area of forest in grid	ha
	The percentage of forest area	PFA	The proportion of forest area in grid	%
	Normalized difference vegetation index	NDVI	It reflects the state of vegetation growth and vegetation coverage	
	Forest contribution to NDVI	FNDVI	The contribution of forest to NDVI in grid	%
Climate	Precipitation	PRE	Annual average precipitation	mm
	Evaporation	E	Annual average evaporation	mm
	Radiation	RA	Annual average radiation	MJ/m^2^
	Temperature	T	Annual average temperature	℃
	Relative humidity	RH	Annual average relative humidity	%
Landscape fragmentation indices	Aggregation index	AI	Connectivity and aggregation among patches of each landscape type	%
	Landscape division index	LDI	It reflects the degree of separation of patches in the landscape	
	Landscape shape index	LSI	It measures the complexity of a shape	
	Patch density	PD	It can reflect the heterogeneity and fragmentation of the landscape	#/100 ha
	Shannon's diversity index	SHDI	It is used to measure the diversity and heterogeneity of landscape types

#### Exploring influential paths of LUCC, landscape and climate factors on soil erosion

**Figure 3:**
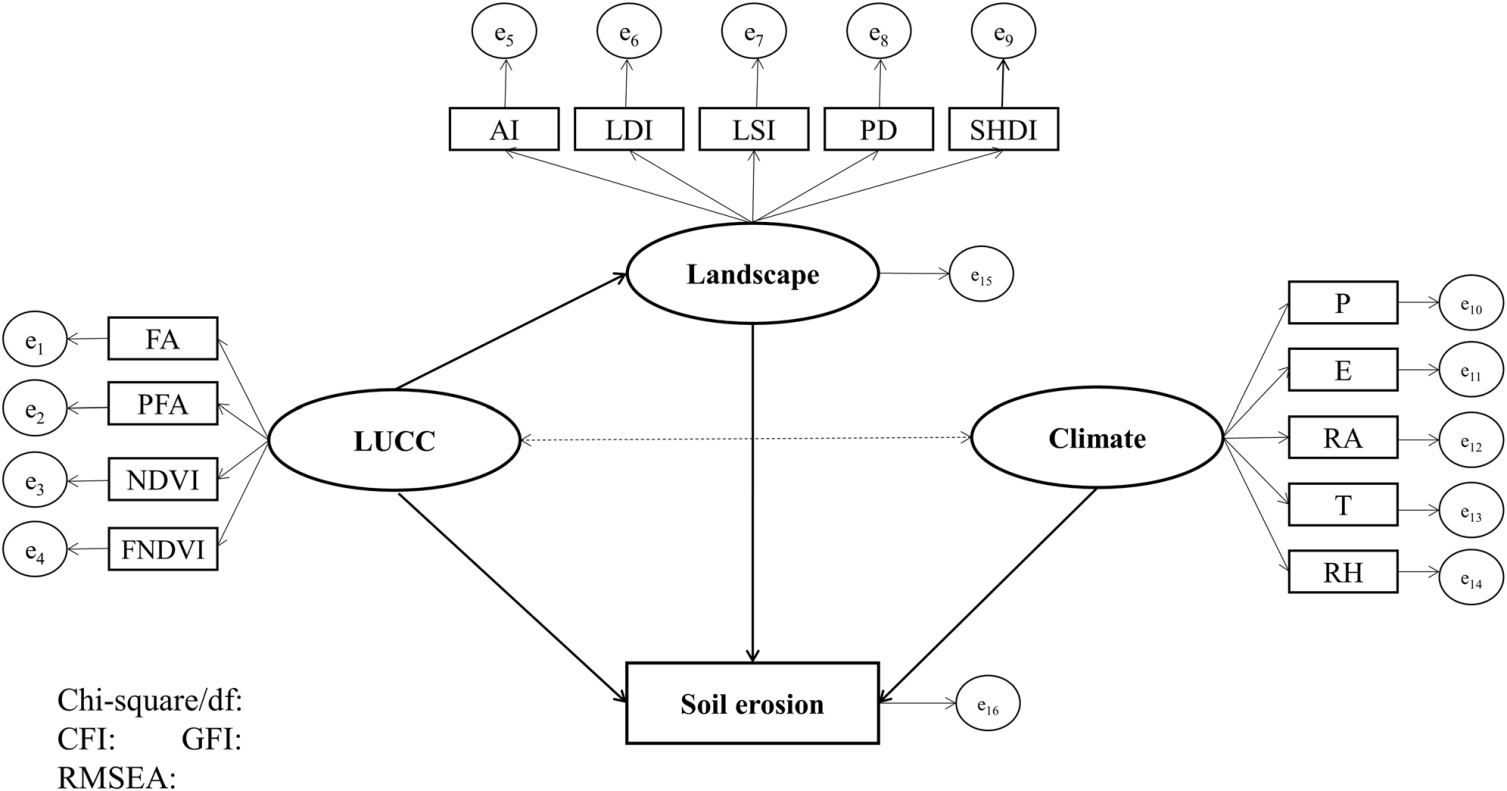
The hypothetical SEM in the research, rectangles represent the observed variables, ellipses stand for the latent variables and circles is the random errors, respectively.

The logical relationships and complex influential paths between variables was verified by using the SEM which is a multivariate statistical method^49^. SEM first requires a hypothetical model of the theory in conjunction with previous studies, which expresses the “cause and effect” relationship between the observed variables or the latent variables in the form of a structural equation^50^. After the equations are formulated, the expected covariance matrix (based on the specified model) is generated and compared with the observed covariance matrix (based on real data)^51^. Model fit tests are then used to determine if the criteria are met between the matrices^47^. The index of chi-square and degrees of freedom (Chi-square/df < 5), the goodness of fit index (GFI > 0.9), comparative fit index (CFI > 0.9), and root mean square error of approximation (RMSEA < 0.1) were applied to test the reliability of the results^52,53^. The hypothetical model in this study is shown in Fig. 3, and more details about the modeling, validation and optimization of SEM could be found in the article of Lam and Maguire, Qiu et al.^47,51^.

All the calculation of RULSE model (including these factors) was performed on the software platform of ArcGIS 10.5 (https://www.esri.com). It was worth noting that all of our raster data were defined with the UTM projection system, in addition to the uniform resolution set to 30 × 30 m before the calculation, respectively. All the statistical analysis in this paper were analyzed in R studio 3.5.0 (including corrplot, ggplot2, lavaan and semPlot packages), and our mapping was done in Origin 2017 and R.

## Results

### Variation of LUCC in Xiushui watershed

**Figure 4:**
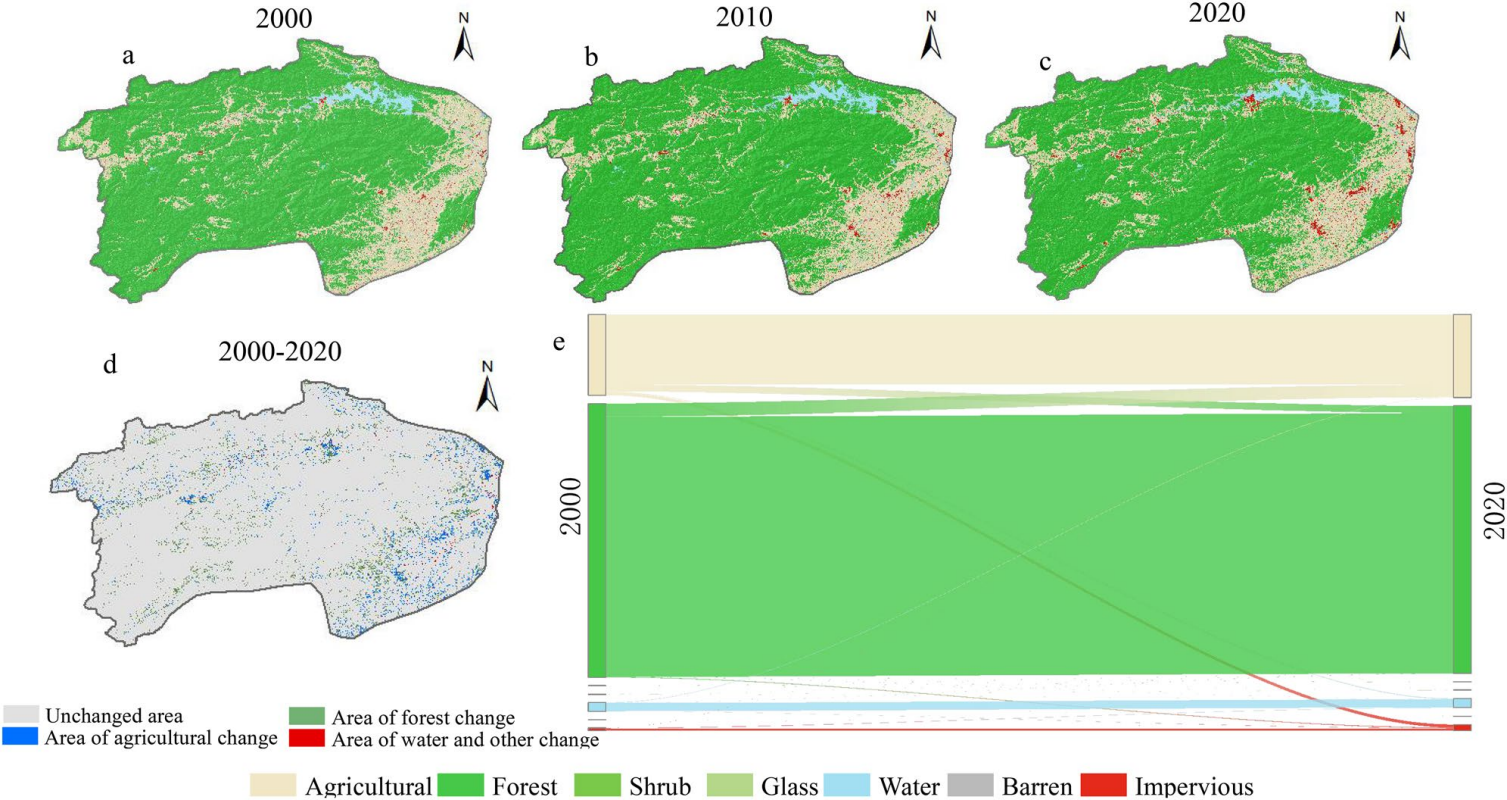
The spatial distribution and variation of LUCC (**a–d**) and the transformation mulberry map of each land use type (**e**) in XSW from 2000 to 2020.

Combining with the “Chinese Classification Criteria of Current Land Use” (GB/T21010-2017) and study objectives, the study area was divided into seven types: agricultural land, forest, shrub, grass, water, barren and impervious. The spatial distribution of land use/cover in XSW from 2000 to 2020 was illustrated in Fig. 4a–c. During the observation period (2000–2020), forest (10.99 ± 0.16 thousand km^2^, mean ± SD) and agricultural (3.27 ± 0.09 thousand km^2^) in XSW were two primary types of LUCC due to their together accounted for more than 90% of the total area, which indicated the abound of the forestry and agricultural resource of the region. In the map of spatial distribution, the forest was located in the southwest and north of XSW at high altitude (the Makufu Mountains and the Jiuling Mountains), and the trunk of Xiushui river was nurtured between the two ranges. In addition, the agricultural land was mainly distributed in the southeast of XSW, which was adjacent to Poyang Lake. (Figs. 1 and 4a–c).

On the temporal scale, Fig. 4a–c showed that the most pronounced change was the impervious, mainly distributed in the southeast of XSW and around the water, which was increasing during the observation period (the ratio of impervious to total area were 2000: 0.76%, 2010: 1.04% and 2020: 1.71%, respectively). From 2000 to 2020, agricultural, water and impervious increased 103.85 km^2^, 19.77 km^2^ and 139.45 km^2^ or by 3.18%, 5.58% and 123.28%. Conversely, forest, shrub, grass, and barren showed a downward trend, with the highest decrease in the area was forest (261.53 km^2^) but with the highest decrease in ratio of the area was barren (78.51%), respectively. Overall, the variation of forests and cropland area was the largest because they originally had a large area (Fig. 4e); but the largest proportional changes in area were barren and impervious.

### Analysis of landscape configuration

**Figure 5:**
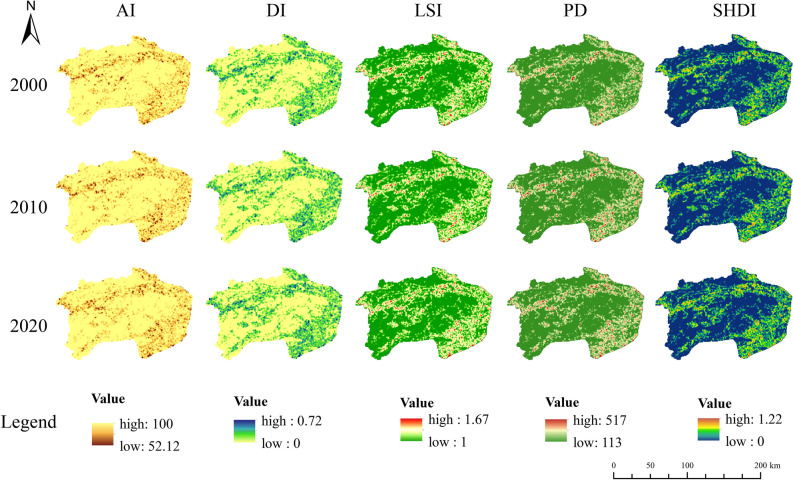
Spatial–temporal distribution of landscape configuration in XSW from 2000 to 2020, which generated in the ArcGIS 10.5 software (https://www.esri.com).

All these landscape indexes (AI, DI, LSI, PD and SHDI) were evaluated in Fragstats (version 4.2) software by using the moving window method and shown in Fig. 5. These indicators can reveal the landscape heterogeneity changes for instance aggregation, separation, fragmentation and so on^26^. Figure 5 clearly showed that the landscape index of forested areas (the north and southwest areas of XSW, Fig. 4) forms two distinct genealogies from the landscape index of agricultural land (around the trunk of Xiushui river and the southeast plain land of XSW, Fig. 1). The maximum and minimum values of AI were 100% and 52.12%, which occurred in the forest area and the southeastern plain zone, respectively. There was no continuous decreasing or increasing trend in AI and LSI during the observation period, and all the mean values of them were above 90% and 1.04 (Fig. 5). However, the DI, PD, and SHDI represent landscape fragmentation had changed somewhat: the mean values of DI in 2000, 2010 and 2020 were 0.056, 0.058 and 0.062, respectively; PD and SHDI during the observation period were 146.36 ± 0.96 (mean ± SD) and 0.087 ± 0.005 with increasing by years, respectively. The results indicated a trend or risk of fragmentation in the landscape.

### Spatial–temporal changes of soil erosion and soil conservation

**Figure 6:**
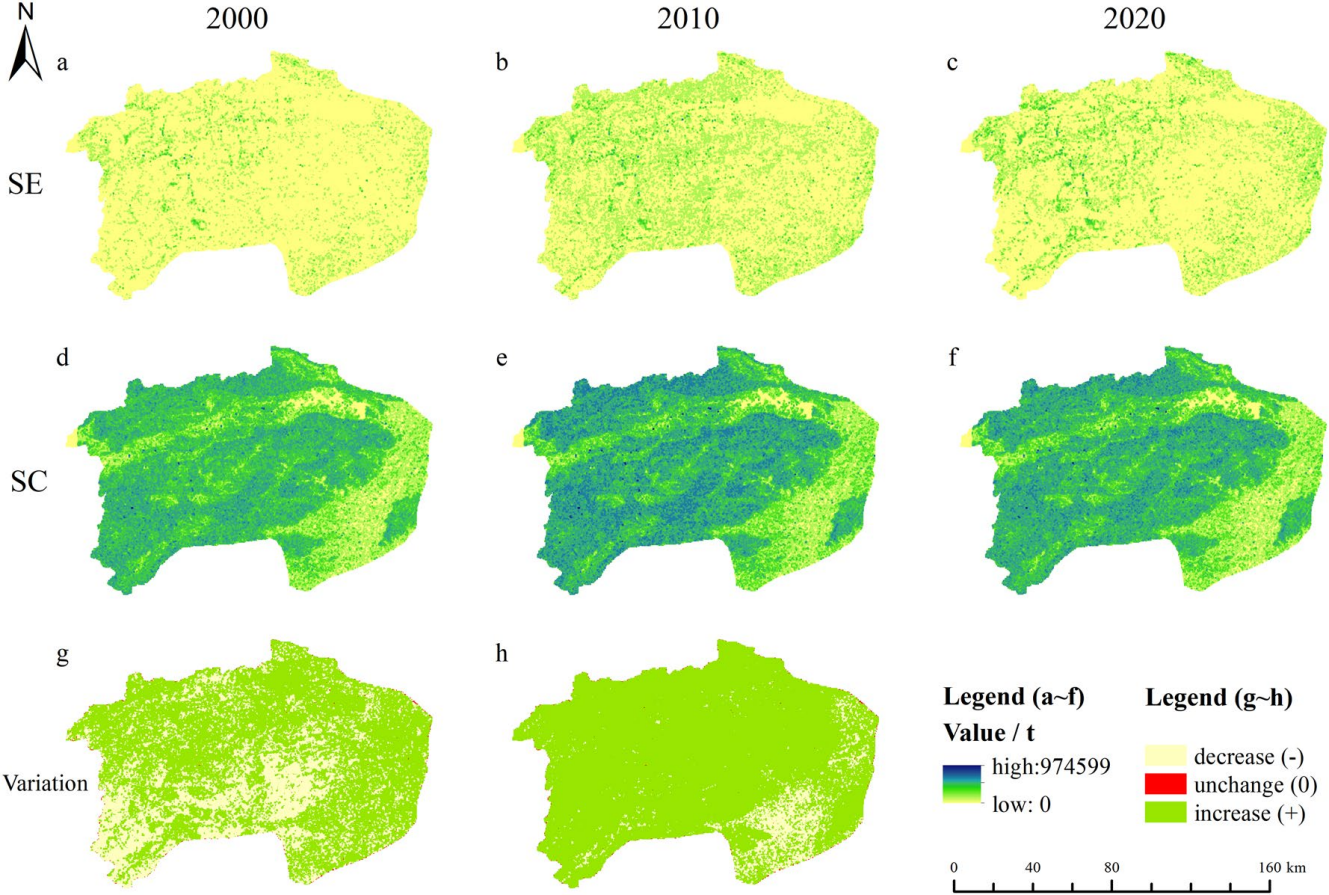
Spatial distribution of soil erosion (SE) and soil conservation (SC) in XSW from 2000 to 2020 (**a–f**) and spatio-temporal variation of SE and SC in the study area from 2000 to 2020 (**g–h**), which generated in the ArcGIS 10.5 software (https://www.esri.com) “green” (or “light yellow”) in (**g**) represents SE (or SC) increase (or decrease) in 2020 than 2000 and “red” in (**g**) or (**h**) represents SE or SC was unchanged from 2000 to 2020, respectively.

The spatial distribution of soil erosion (SE) and soil conservation (SC) in XSW from 2000 to 2020 were simulated based on the RUSLE models (Fig. 6). The areas with severe soil erosion were mainly located in the northwest and southeast of the XSW, with the northwest SE occupying most of the watershed, respectively (Fig. 6a–c). The SE in XSW ranged between 0 and 16,530 t/ha in 2000, between 0 and 21,556 t/ha in 2010, and between 0 and 23,333 t/ha in 2020, and the mean values of them were 2205.27 t/ha, 3414.25 t/ha and 3319.44 t/ha, respectively (Fig. 6a–c). Moreover, the area with the highest risk of soil erosion (darker colored points) occurs near the trunk and tributaries of Xiushui river and mountains (Figs. 6 and 1), which implies that SE around the river and mountains would be a hot spot for control measures.

The three periods of SC exhibited similar spatial distribution characteristics (Fig. 6d–f). The higher value of the SC was clumped within the northern and southwestern area (Fig. 6d–f), where there was high forest cover (Fig. 4). There was no continuous decreasing or increasing trend in SC during the observation period, and the mean values of them were 56,118.47 t/ha, 88,775.99 t/ha and 72,108.367 t/ha.

Spatial–temporal variation of SE and SC in XSW from 2000 to 2020 were illustrated in Fig. 6g, h. More than two-thirds of the SE in the XSW was increased in 2020 compared with 2000 (Fig. 6g), mainly distributed in the trunk and tributaries of Xiushui river and the cultivated land in the southeast of XSW. The remaining one-third was the area of increased SE in 2020 had a common feature that most of them were distribution area of forests (Fig. 4). The distribution of SE was consistent with the results of landscape fragmentation, which suggested that there was a risk of increased SE from landscape fragmentation (Figs. 5, 6g). The area with increased SC in 2020 accounts for more than 90% of the XSW, which matched the results in Fig. 6g.

### Identification of dominant factors for soil erosion

**Figure 7:**
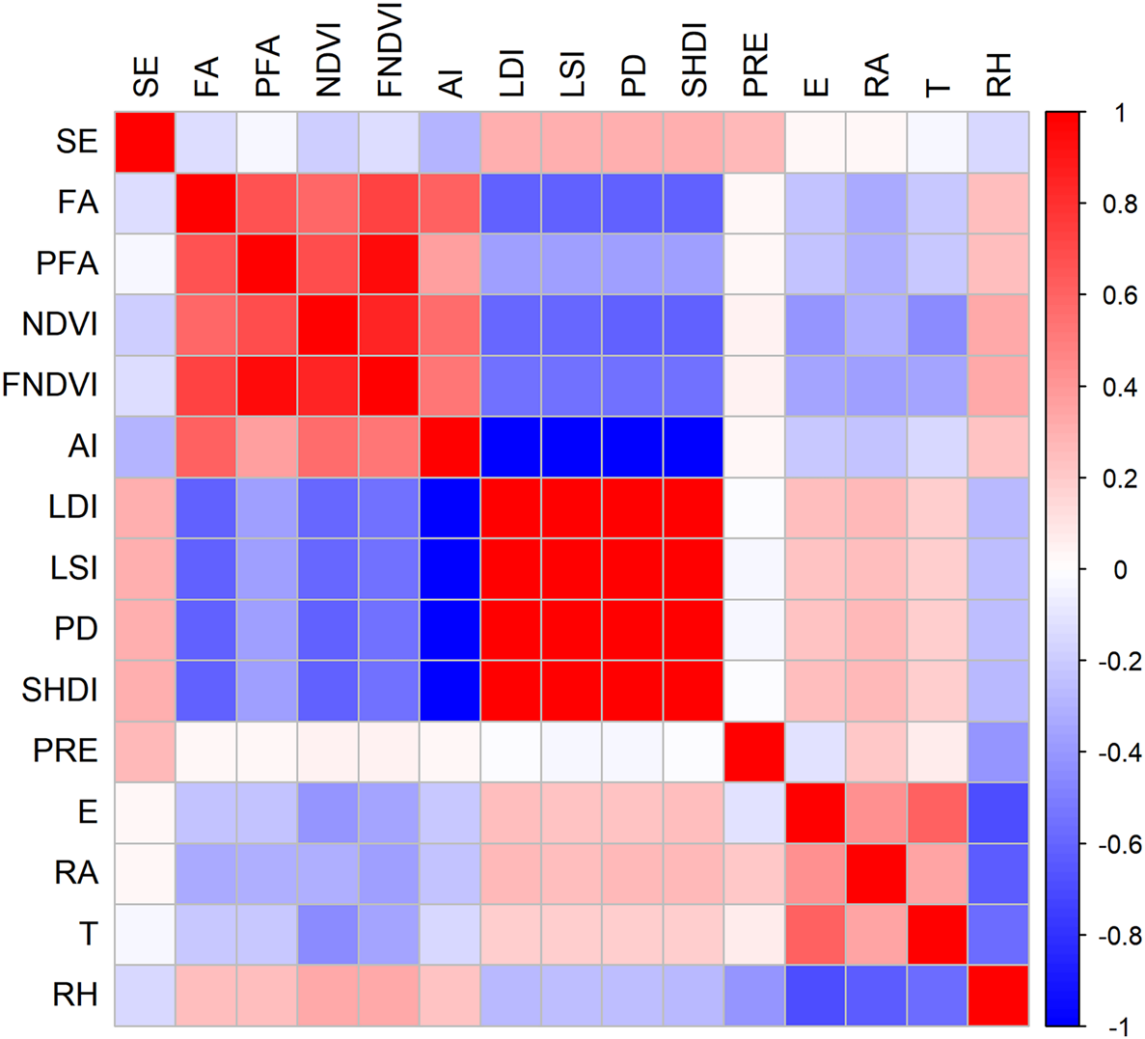
Correlation of soil erosion with LUCC factors, landscape fragmentation factors and climate factors generated in the R (version 4.2.1) (https://www.r-project.org/). LUCC factors included forest area (FA), percentage of forest area (PFA), NDVI and forest contribution to NDVI (FNDVI); landscape fragmentation factors including aggregation index (AI), landscape division index (LDI), landscape shape index (LSI), patch density (PD) and Shannon's diversity index (SHDI); climate factors including precipitation (PRE), evaporation (E), radiation (RA), temperature (T) and relative humidity (RH), respectively. Red (blue) represents a significant positive (negative) correlation and white represents no significantly correlation between the two; the deeper the color, the stronger the effect, respectively.

Evaluation of the relationship between LUCC metrics, landscape fragmentation index, climate metrics and SE using Pearson correlation analysis revealed that there was a significant correlation (*P* < 0.05) among them during the observation period, while the correlations were illustrated in Fig. 7. The LUCC factors dominated by forests (FA, PFA, NDVI and FNDVI) had a significant negative correlation with SE (*P* < 0.01), and correlation coefficients ranging from 0.02 to 0.18 while the strongest correlation was NDVI (Fig. 7). The landscape indices except for AI had a significant positive correlation with SE (*P* < 0.01) and the mean value of the correlation coefficient was 0.31 (Fig. 7). The results indicated that the aggravation of landscape fragmentation would increase the risk of SE. There was a significant positive correlation between rainfall and SE (*P* < 0.01), and the correlation coefficient was 0.27, respectively. Moreover, the remaining climate factors (E, RA, T and RH) had a weak negative effect on SE (Fig. 7).

**Figure 8:**
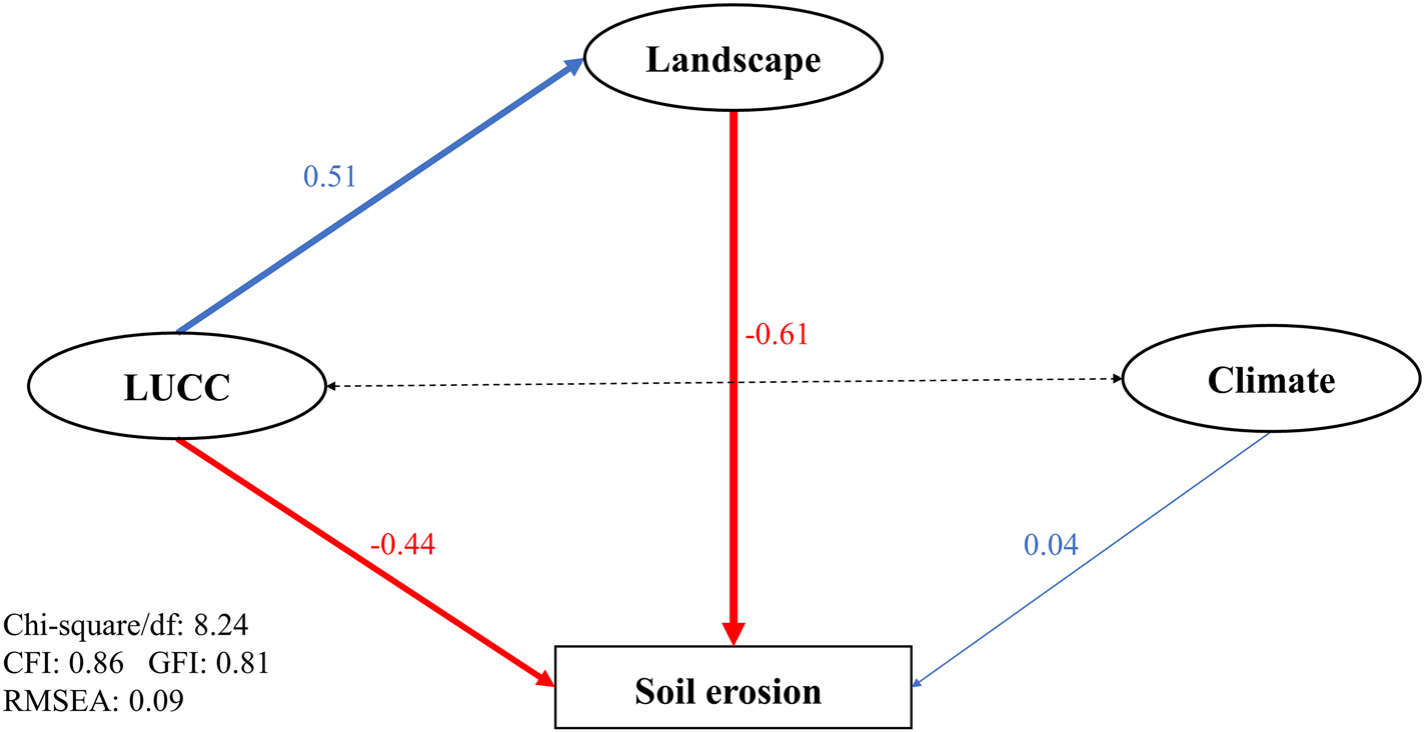
Results from structural equation modeling (SEM) on the soil erosion. Ellipses represent the latent variables (LUCC, landscape and climate), rectangles represent the observed variables (SE); FA, PFA, NDVI, and FNDVI were binned into the latent variable LUCC, whose most relevant observed variable was FNDVI (*R*^2^ = 0.97); AI, LDI, LSI, PD, and SHDI were binned into the latent variable Landscape, which was positively correlated with AI (*R*^2^ = 0.97); climate was a latent variable consisting observed variables (PRE, E, RA, T, and RH) which was positively correlated with PRE (*R*^2^ = 0.41), respectively. Red numbers or lines indicate the negative effects between the variables, blue numbers or lines indicate the positive effects between the variables; unidirectional arrows representing paths between variables and bidirectional arrows indicating correlations; the solid line indicates that the path is significant (*P* < 0.05), the dashed line indicates that it is not significant (*P* > 0.05) and the thickness of the line represents to the effect size, respectively.

The Chi-square/df, GFI, CFI and RMSEA were applied to test the reliability of the results. The SEM had basically acceptable fitness due to RMSEA in the interval [0.08,0.1]^54^, though CFI and GFI did not meet the requirement of greater than 0.9^46^. Moreover, the model had a high *R*^2^ (0.85), which meant that the model could explain 85% of the variability in SE. Standardized path coefficients were used to measure the effect between variables in the paths, as they allow direct comparison of the degree of effects among variables measured on different scales^55^. As shown in Fig. 8, three latent variables (LUCC, landscape and climate) had direct interactions on SE. Specifically, LUCC and the magnitude of landscape aggregation had a negative effect on SE with path coefficients of -0.61 and -0.44, respectively (*P* < 0.01); climate factors also had a directly driving effect on soil erosion with a path coefficient of 0.04 (*P* < 0.01). In addition, the path of LUCC to landscape aggregation was 0.51 (*P* < 0.01), which indicated the landscape aggregation was also influenced by the forest-dominated LUCC factors.

## Discussion

### Spatial and temporal variation of soil erosion

LUCC was identified as one of the most significant factors affecting SE and SC, because shifting land cover could result in lost arable land on-site and silting off-site rivers^56^. The results of this research showed that LUCC could have a directly effect on soil erosion (Fig. 8), which was consistent with Liu et al.^9^ who obtained different SE results by simulating LUCC in different scenarios. In addition, we also found that the value of SE under forest cover was lower than agricultural land for three years of the observation period (Table 3). This result indicates that afforestation can effectively reduce the risk of SE, especially in poor areas of SE^10,57^. However, the higher value of the SE was not always clumped within arable land, but also occur in forested areas (Fig. 6a–c). This indicates that soil erosion was influenced by other factors. In the research, most forest areas located in mountainous, where the high length and slope increased the risk of soil erosion^29^. Under certain topography, the slope length factor can cause 60% effect on soil erosion^30^. Overall, soil erosion heat zones were mainly distributed in the cultivated land around Xiushui river (Figs. 3 and 6a–c), which was similar to the preceding research^44,56^.

**Table 3:** The mean value of SE under different LUCC from 2000 to 2020 (unit: t/ha).

Time/LUCC	Agriculture	Forest	Shrub	Glass
2000	5067.00	1449.02	4958.42	4866.09
2010	7836.37	2297.05	10,025.27	5717.44
2020	9565.71	1553.86	10,070.69	6393.49
Mean	7489.69	1766.64	8351.46	5659.01

In the dimension of time, the average values of SE were 2205.27 t/ha, 3414.25 t/ha and 3319.44 t/ha from 2000 to 2020, respectively (Fig. 6a–c). The increase in SE could be caused by a dramatic increase in rainfall^13^. The annual rainfall in 2000, 2010 and 2020 were 1492.69 mm, 2028.43 mm and 1749.07 mm, compared to the average rainfall for multiyear (1681 mm)^36^, 2010 exceeds 374 mm. It is not difficult to understand why the mean value of SE in 2010 was much higher than in 2000 and 2020, because different rainfall conditions bring runoff could lead to an exponential increase in sediment transport^28^. Meanwhile, evidence of increased soil erosion due to dramatic increases in rainfall can be found in our potential soil erosion data: the potential soil erosion in 2010 was higher 16–33 kt/ha than 2000 and 2020.

Another noteworthy phenomenon in this study was that the difference in rainfall between 2010 to 2020 (∆rainfall_2010–2020_: 279.36 mm) and 2020 to 2000 (∆rainfall_2020–2020_: 256.38 mm) was similar, but the difference in SE between the same years was huge (∆SE_2010–2020_: 94.81 t/ha, ∆SE_2020–2000_: 1114.17 t/ha), which may suggest that there were other factors that offset the SE of increased rainfall. SE is positively correlated with precipitation and negatively correlated with forested area^22,58^, and when the *K*-factor and *LS*-factors are relatively fixed in the study area, the only factors that could offset the effect of rainfall are the *P*-factor and the *C*-factor (Eq. 1)^16^. The *P*-factor affects SE via the area differences in land types^16^, while the difference in forest area between 2010 to 2020 was 281 km^2^; also, the vegetation growth status (NDVI) affects the SE by affecting the *C*-factor^59^, while the difference in NDVI between 2010 to 2020 was 0.078, and the difference in forest area and NDVI between 2020 to 2000 were −261 km^2^ and −0.068, respectively. The combination of reasons leaded to a situation where the difference in rainfall was slight but the difference in SE was huge, while it suggested that except forest area, forest quality (e.g. NDVI reflecting vegetation growth status) has great potential to reduce SE.

### The driving factors of soil erosion

The landscape factor had a negative effect on SE in the SEM of this research (−0.61, *P* < 0.01) (Fig. 8). First of all, it was clear that the landscape latent variable in this study was positively related to AI and had a negative effect with LDI, LSI, PD, and SHDI, respectively. This suggested that the more fragmented the landscape, the more severe the soil erosion. It was consistent with Mitchell et al.^60^ who found that landscape fragmentation led to lower water yield and increased soil erosion. Meanwhile, Bai et al.^33^ has also established a significant positive correlation between AI and soil conservation in the Taihu watershed. Fragmentation of the landscape cut off the original material and energy flows and increased the "edge effect" between sub-ecosystems^61^. These "edges" had different material and energy flows than the "center", which resulted in the increase of landscape heterogeneity^61^. Besides, the increase in forest "edges" could lead to increased soil erosion by rainfall directly striking the topsoil and forming runoff that washed the soil surface^62^; the change in cohesion between interfaces occurred at the "edge" of the arable land, which exacerbated the export of nutrients and soil particles^62–64^. The mechanism between landscape fragmentation and increased soil erosion is still a blank area that is interesting and worthy of further study, but landscape fragmentation led to the increase of soil erosion and the decrease of ecosystem services which was consistent with Hu et al.^26^, Harper et al.^61^ and this research.

The landscape factor was the most dominant pathway affecting soil erosion in the SEM (−0.61, *P* < 0.01) (Fig. 8), and its standardized path coefficients was greater than those of LUCC and climate factors. This was similar to the results of Bai et al.^33^ who founded that landscape configuration (landscape fragmentation) played a stronger role than landscape composition (LUCC). However, the ecological services assessment in terms of hydrology founded that LUCC was more important^65^. This phenomenon was attributed to the heterogeneity of the landscape and the differences in the scale of the study area^33,65^. It was also one of the reasons for the lower climate factor path coefficients. We also found that rainfall had a significant positive effect on soil erosion (Fig. 7) which consistent with most previous studies^22,29^. However, the causality of the SEM was not exactly equivalent to correlation^51^, and the effect of rainfall on soil erosion might be underestimated due to the mixing of multiple climate elements. Additionally, our results were consistent with Hu et al.^26^ who found that the contribution of the landscape index was greater than that of rainfall and evaporation by principal component analysis (PCA) in Dongting Lake watershed.

### Limitations and future research

In this research, the RULSE model was utilized to assess soil erosion in XSW. Although the model is a widely used tool in the estimation of regional and global annual SE^7,8,50^, there was still potential room for improvement as some empirical parameters was included in the model^31^. Additionally, large-scale spatial applications typically used coarse data, which was not compatible with the local scale on which the model was parameterized^31^. Although the estimation of soil erosion base on the RULSE model was shown to be appropriate, it is necessary to optimize the model parameters, and more accurate results requires high-resolution data sources^26^.

Lam and Maguire suggested that comprehensive multifactorial assessments would be vital and feasible, and that the conclusions of these impact assessments (SEM) would be more convinced than single-factor analyses^51^. In the research, we comprehensively analyzed the effects of LUCC, landscape fragmentation and climate on SE. The result of this study provides evidence on soil erosion and its driver metrics, which could also provide a reference for similar studies within and beyond China. However, the contribution of rainfall to soil erosion was often underestimated on an long term and large spatial scale, and soil erosion due to extreme rainfall would account for a large proportion of total soil erosion throughout the year^13,44^. Therefore, it is a great challenge to downscale soil erosion due to extreme rainfall on short calendar periods. In addition, the intrinsic influence mechanism between landscape fragmentation and soil erosion at the micro level, which is an interesting blank area and should be the focus of future studies.

## Conclusions

Soil erosion in the XSW was effectively assessed based on the RULSE model, and the paths of the main drivers of soil erosion were investigated by statistical analysis (correlation analysis and SEM). Our results showed that there was no continuous increase or decrease trend on the SE of XSW due to the impact of rainfall; soil erosion was directly driven by LUCC factors (dominated by NDVI), landscape fragmentation and climate factors, where the drivers of LUCC and landscape fragmentation were greater than the underestimated climate factors. This suggested that increasing the area of forests was an effective way to reduce soil erosion, but improving the quality of forests (NDVI, canopy closure, structure) should be received greater attention. Meanwhile, rational planning of land types and reduction of landscape fragmentation could reduce the risk of soil erosion. This study provides a useful reference for maintaining the sustainable development of fragile ecological zones.

## Data Availability

The datasets used and/or analyzed during the current study available from the corresponding author on reasonable request.
